# Assessment of knowledge, attitudes, practices, and barriers to evidence-based practice (EBP) among healthcare providers at Port Said: an exploratory sequential mixed method study

**DOI:** 10.1186/s12909-025-08398-8

**Published:** 2025-12-19

**Authors:** Menna Alaa El-Khouly, Huda Ramadan Taher, Mohammed Yasser Abboushi Abboushi, Abrar Elsayed Elgayar, Shady Mohamed Lskaan, Ahmed Mahmoud Mohamed Abd El-Wahab, Nesrine Saad Farrag, Nesreen F. Ibrahim

**Affiliations:** 1 Faculty of Medicine, Port Said University, Port Said city, Egypt; 2 Head of public health, preventive and social medicine, Department of Public Health Community, Environmental and Occupational Medicine, Faculty of Medicine, Port Said University, Port Said city, Egypt; 3 Lecturer of public health, preventive and social medicine, Department of Public Health Community, Environmental and Occupational Medicine, Faculty of Medicine, Port Said University, Port Said city, Egypt; 4 Faculty of Medicine, Port Said University, 14 Ahmed El Nagar st, Mansoura, Dakahlia Egypt

**Keywords:** Evidence-based practice, Egypt, Mixed method, Healthcare providers

## Abstract

**Background:**

Evidence-based practice (EBP) is the integration of best research evidence, clinical expertise, and patient values in healthcare decision-making. Although EBP is regarded as the gold standard in healthcare systems, influencing policy, education, and practice, the Arab region continues to face significant barriers to the extensive implementation of EBP.

**Methods:**

This was a cross-sectional study in which a mixed-methods approach with an exploratory sequential design was used. A validated questionnaire was developed in the first qualitative phase on the basis of the inductive thematic analysis results. Then, it was used in the quantitative phase. Significance was determined at the 95% confidence level.

**Results:**

The qualitative phase included 37 healthcare providers of all specialties from four hospitals in Port Said city. Ten themes with 35 sub-themes were generated that discussed different aspects of knowledge, attitudes, practices, and barriers to EBP. The quantitative phase included 443 healthcare providers from five hospitals in Port Said City. The majority of the participants revealed good levels of knowledge, positive attitudes, and practices toward EBP. Age, profession, training, and workplace were the determinants of knowledge. Knowledge was the only predictor of attitudes. Workplace, research skills, training, knowledge, and attitudes were predictors of practice. The most common barriers included lack of time, insufficient financial support, and workload.

**Conclusion:**

Despite good levels of knowledge, attitudes and practices, a considerable percentage of the participants had poor knowledge and practices. The study indicates the need for hospitals to include evidence-based protocols and supervise their implementation, and to establish a research department that provides healthcare providers with the needed facilities to conduct research. Additionally, increasing financial support for healthcare systems to improve facilities and providers is as appreciation and encouragement for the application, and more attention should be given to establishing Egyptian guidelines for all specialties. Also establishing EBP center that provide the training services, support research, guide the physician to apply EBP, and ensure the application of EBP will be impactful.

**Supplementary Information:**

The online version contains supplementary material available at 10.1186/s12909-025-08398-8.

## Introduction

Evidence-based practice (EBP) has emerged as a cornerstone of modern healthcare, bridging the gap between scientific research and clinical decision-making. It emphasizes the integration of best research evidence, clinical expertise, and patient values in healthcare decision-making. Globally, it is documented as a vital tool for quality upgrading [1]. 

EBP is defined as the conscientious, explicit, and judicious use of the best current evidence in making decisions about the care of individual patients or populations. This process involves systematically searching, appraising, and applying research findings to clinical practice [1]. 

The World Health Organization (WHO) emphasizes evidence-informed decision-making as essential for improving patient outcomes, reducing healthcare disparities, and achieving sustainable development goals (SDGs) [2]. 

High-income countries have well-established infrastructures for EBP. They invest profoundly in research production, systematic reviews, and clinical guidelines, which are dispersed through organizations such as the Cochrane Collaboration and the National Institute for Health and Care Excellence (NICE) [3]. 

These countries also integrate EBP into the curricula of medical and nursing schools, ensuring that future healthcare professionals acquire the skills needed for critical appraisal, evidence synthesis, and application [4]. 

While Arab countries and Egypt have made outstanding progress in implementing EBP. But challenges related to infrastructure, training, and cultural acceptance remain. Addressing these gaps is critical for ensuring sustainable advances in patient care and public health outcomes [2]. 

However, the Arab region continues to face significant barriers to the extensive implementation of EBP. These include limited funding for research, lack of access to international journals, variability in healthcare quality, and insufficient continuing education chances for healthcare providers [5]. 

Additionally, cultural and administrative barriers sometimes limit the acceptance of EBP in clinical settings, where traditional practices and hierarchical decision-making often dominate over research-based recommendations [6]. 

Egypt, as one of the largest Arab countries, presents a unique case study on the adoption of EBP. With a rapidly growing healthcare system serving more than 110 million people, the integration of evidence-based approaches is vital for improving outcomes and improving resource allocation [7]. 

National health policies in Egypt have increasingly aligned with evidence-based approaches. For example, the Ministry of Health and Population collaborate with international organizations such as the WHO to develop guidelines for disease control, maternal and child health, and infection prevention [8]. 

The COVID-19 pandemic has increased the importance of EBP in Egypt, as clinicians and policymakers need to rely on rapidly evolving global evidence to guide prevention strategies, vaccination policies, and clinical management protocols [9]. 

Despite this progress, Egypt continues to face difficulties in fully institutionalizing EBP. These include limited financial investment in research, inadequate training opportunities for practicing clinicians, and challenges in disseminating guidelines to rural and underserved areas [10]. 

Surveys conducted among Egyptian healthcare providers suggest that while there is a positive attitude toward EBP, there are knowledge gaps and a lack of confidence in critical appraisal skills. This reflects the need for continuous professional development and mentorship programs [11]. 

As no study has explored the knowledge, attitudes, and practices of EBP among healthcare providers in Port Said city, this study aimed to help improve the quality of healthcare and medical outcomes by developing and validating a questionnaire to assess the knowledge, attitudes, and practices regarding EBP among healthcare providers in Port Said city and detecting the barriers to its application after these subjects were explored. Then, we measured them quantitatively and identified their associated factors.

## Methods

### Study design

The study was conducted between January 2025 and September 2025 through two phases:

#### Phase 1

This study was conducted through a mixed methods approach with an exploratory sequential design. It involves a qualitative data collection and analysis approach through semi-structured interviews. One-on-one interviews via face-to-face meetings with healthcare providers in Port Said City were used to generate items, which were drafted into an initial questionnaire, validated by public health experts via the content validity index (CVI), pilot tested among a small group of healthcare providers, and assessed for reliability via Cronbach’s alpha.

#### Phase 2

A cross-sectional analytic study was conducted using a structured interview questionnaire that was developed and validated on the basis of the findings of the qualitative approach.

Therefore, Clinical trial number is not applicable.

### Study setting and population

The study was conducted in Port Said City’s primary, secondary, and tertiary healthcare facilities. The study population included physicians, dentists, pharmacists, nurses, physiotherapists, and health technicians working in these hospitals.

#### Inclusion criteria

Any registered healthcare professional (physicians, nurses, pharmacists, physiotherapists, dentists, or health technicians) who has direct contact with patients and has been practicing for at least 6 months.

#### Exclusion criteria

Those who are not present during the data collecting period, trainees or recently hired employees, administrative staff who are not actively involved in patient care or healthcare decision-making, and those who refuse to participate or do not finish the questionnaire.

### Sampling

#### Sample size

❖ For the qualitative phase:

We collected data until we reached the saturation point at which we were not receiving new information. An iterative process of data collection and analysis ensures data saturation. The research team performed preliminary coding following each set of responses and contrasted recently developed concepts with previously recognized themes. To record whether new concepts were introduced in each successive interview or response, a saturation table was kept. Data collecting continued until no new themes emerged in the final two rounds of analysis, indicating information redundancy. The total sample included 37 interviews.

❖ For the quantitative phase:

- The sample size was calculated via Epi Info Version 3 at the 95% confidence interval (CI) level, with a margin of error of ± 5% and 80% power. A previous study conducted in Tanta, Egypt, reported that 10.5% of physicians had good knowledge of EBM, and the calculated sample size was 420 after adding 10% of the calculated sample size to avoid dropout [12].

#### Sampling procedure

For the qualitative phase, a purposive sampling approach was used to consider and reflect the diversity among healthcare providers.

For the quantitative phase, a multistage sampling approach was used to select healthcare providers from different hospitals. In the first stage, hospitals were stratified according to type (tertiary, secondary, and primary care), and a random sample of hospitals was chosen from each category to ensure representativeness. In the second stage, departments relevant to the study, such as emergency, surgery, pediatric, obstetric, and intensive care departments, were identified within the selected hospitals, and a random selection of departments was made. In the third stage, all healthcare providers, including doctors, nurses, and technicians, were included in the study.

### Study tools

#### For the qualitative phase

A structured interview guide was developed (Appendix 1) after the literature was reviewed from another study done on evidence-based medicine therefore some edits had been done to match our objectives [13]. One-on-one interviews via face-to-face meetings were conducted. It covers four main categories: knowledge about EBP, attitudes toward EBP, the practice of EBP, and barriers to implementing EBP.

#### For the quantitative phase

The structured questionnaire that was developed and validated on the basis of the results of the qualitative phase was used to assess the knowledge, attitudes, and practices of EBP on a larger scale.

The questionnaire was validated by three independent academic experts (two professors and one lecturer) to ensure its relevance to the study objectives, clarity, and appropriateness. The scales showed an excellent content validity index, as all the items were rated as highly relevant and clear by all the experts, and some modifications were made. Each expert evaluated the relevance of every questionnaire item using a 4-point scale ranging from 1 (not relevant) to 4 (highly relevant). Item-level content validity indices (I-CVI) were calculated as the proportion of experts rating each item as either 3 or 4. With three experts, an item achieves an I-CVI of 1.00 only when all experts rate it as relevant. All items met this criterion, resulting in I-CVI values of 1.00 for every item. The scale-level content validity index (S-CVI), calculated using the average method (S-CVI/Ave), was therefore 1.00, indicating complete agreement among experts regarding item relevance.

A pilot study of 50 participants was subsequently conducted to ensure the clarity of the questionnaire and the feasibility of the study. The pilot indicated that all the questions were clear and accepted. The results of the pilot study were included in the study’s results, as no further modifications were made.

The questionnaire consisted of several sections. The first section collected sociodemographic data, including age, sex, degree of education, years of experience as a general practitioner and as a specialist, hospital, profession, specialty, evidence-based practice (EBP) courses or training, and frequency of reading journals. This section also included self-rating questions to measure research experience and critical appraisal skills. The second section measured knowledge about EBP and comprised five questions, of which three were false and two were true. The third section assessed attitudes toward EBP through 24 questions on a three-point Likert scale, whereas the fourth section measured the practice of EBP via five yes/no questions. Finally, the questionnaire explored barriers to the application and learning of EBP, such as time limitations, workload, lack of resources, lack of guidelines, and cultural aspects.

### Qualitative analysis

Inductive thematic analysis was used to provide a deeper understanding of the findings and provide more explanations. It aimed to identify themes from the data. It involves becoming familiar with the data, coding, generating themes, reviewing and refining themes, naming and defining the themes, and interpreting the findings.

### Quantitative analysis

The data were analyzed via SPSS Statistics for Windows, version 29.0, and managed by Microsoft Office 2019. The Kolmogorov–Smirnov test was used to assess normality. The numerical variables were categorized according to the median. All categorical variables are presented as frequencies and percentages in suitable tables or figures. The chi-square test was used to evaluate associations between categorical variables, and Fisher’s exact test was used instead of the chi-square test when more than 20% of the cells had expected frequencies < 5. Statistical significance was determined at the 95% confidence level (i.e., differences were considered significant if the P value was ≤ 0.05). There were no missing data. Binary logistic regression was performed to determine the independent significant predictors of good adherence among participants.

*For the knowledge score*, this section included five questions, three false and two true. Each correct answer was encoded as True = 1 and False = 0, and the false answers were reverse coded as True = 0 and False = 1. The total score was 5, and the hypothetical median was 3. Therefore, the responses were categorized as “good knowledge” if their total score was above the median and “poor knowledge” if their score was at the median or below. The scale showed high content validity (S-CVI (scale content validity index) = 1, S-CVI universal agreement = 1) and questionable internal consistency (Cronbach’s alpha = 0.62).

*For the attitude score*, this section included 24 questions on a three-point Likert scale. Each positive statement was encoded (Disagree = 1, neutral = 2, Agree = 3), and the negative statements were encoded reversely. The total score ranged from 24 to 72, and the hypothetical median was 48. Therefore, the responses were categorized as “positive attitudes” if their total score was above the median and “negative attitudes” if their score was at the median or below. The scale showed high content validity (S-CVI (scale content validity index) = 1, S-CVI universal agreement = 1) and good internal consistency (Cronbach’s alpha = 0.82).

*For the practice score*, this section included five yes or no questions. Each answer was encoded as yes = 1 and no = 0. The total score was 5, and the hypothetical median was 3. Therefore, the responses were categorized as “good practice” if their total score was above the median and “poor practice” if their score was at the median or below. The scale showed high content validity (S-CVI (scale content validity index) = 1, S-CVI universal agreement = 1) and good internal consistency (Cronbach’s alpha = 0.747).

## Results

### Study phase 1: qualitative findings

This phase included 37 healthcare providers of all specialties from four hospitals in Port Said city. The mean age was 33.08 ± 7.64 years. The mean years of experience was 8.86 ± 7.92. Most of them were females (59.5%), graduated from the faculty of medicine (48.6%), had a bachelor’s degree (67.6%), and never had an EBP or EBM course or training (94.6%) Table 1. 

**Table 1:** Sociodemographic data and characteristics of the study participants in the qualitative phase (*n* = 37)

Sociodemographic data		Frequency	%
**Age**	Mean ± SD		33.08 ± 7.64
**Years of Experience**	Mean ± SD		8.86 ± 7.92
**Gender**	Male	15	40.5
	Female	22	59.5
**Specialty**	General Practitioner	5	13.5
	Pharmacist	5	13.5
	Nursing	7	18.9
	Physiotherapist	3	8.1
	Cardiologist	2	5.4
	Maxillo-facial surgeon	2	5.4
	Clinical pharmacist	2	5.4
	Others*	11	29.7
**Previously attended an EBP course**	No	35	94.6
	Yes	2	5.4
**Educational Degree**	Bachelor	25	67.6
	Master’s Degree	4	10.8
	Fellowship	1	2.7
	Doctoral Degree	7	18.9
**Total**		37	100

Table 2 shows that ten themes with 35 subthemes were generated in this study that discussed different aspects of knowledge, attitudes, practices, and barriers to EBP.

**Table 2:** Summary of the generated themes and subthemes (*n* = 37)

Theme	Subtheme
**Perception of the EBP concept**	EBP aspects
	EBP knowledge sources
	Difference between EBP and EBM
**Attitude of EBP**	Saving provider’s resources
	Positive Self-effects
	Better patient healthcare
	Improving the patient’s psychology
	Saving patient resources
	EBP benefits for the healthcare system
	Improving the doctor‒patient relationship
	Motivation and intrinsic interest in EBP
	EBP is more beneficial in specific cases
**Benefits of EBP involvement in the medical curriculum**	Improve the new medical generation experience
	Improve the healthcare system
	Improve the patient’s health
	EBP more useful for postgraduates
**Determinants of reaching the needed information**	General facilities
	Self-efforts
	Hospital facilities
	Hospital barriers
	Financial Burden
**Research experience**	limited to publication
	General experience
	Sources of research experience
**Critical appraisal skills**	Bases of critical appraisal
	Causes of Limited Critical Appraisal Experience
**Application of EBP**	Bases of EBP application
	Situations of EBP application
	No application of EBP
**Barriers to implementing EBP**	patients-related barriers
	communication issues
	Provider-related barriers
	hospital policies and resources
**Barriers to learning EBP**	Provider related
	National issues
**Recommendations for improving EBP practice**	

*Others: One participant for each of the following specialties: dermatology, general surgery, cardiothoracic surgery, plastic surgery, nephrology, vascular surgery, ophthalmology, toxicology, pediatric, and neurosurgery radiology

Finally, we developed and validated a structured questionnaire on the basis of the previous results. It included five sections, as demonstrated in Table 2^2^ in the supplementary material.

Study phase 2: quantitative findings

The quantitative phase included 443 healthcare providers from five hospitals in Port Said City. The majority of the participants were 23 years of age or younger (53.3%), were female (65.7%), had been employed for five months or less (53.3%), were nurses (60.9%), did not have courses or training in evidence-based practice (EBP) before (67%), read journals in demand (49.2%), had a bachelor’s degree or diploma (87.1%), and were working at Hospital 1 (39.3%), as shown in Table 3^3^.

**Table 3:** Sociodemographic characteristics of our population (*N* = 443)

Variables	Frequency (%)		
**Age**	≤ 23 years	236 (53.3)	
	> 23 years	207 (46.7)	
**Gender**	Male	152 (34.3)	
	Female	291 (65.7)	
**Experience Period**	≤ 5 months	236 (53.3)	
	> 5 months	207 (46.7)	
**Profession**	Doctor	109 (24.6)	
	Nursing	270 (60.9)	
	Pharmacist	59 (13.3)	
	Dentist	4 (0.9)	
	Physical therapist	1 (0.2)	
**EBP courses or training**	Yes	146 (33)	
	No	297 (67)	
**Frequency of reading journals**	Regular	94 (21.2)	
	Occasionally	131 (29.6)	
	On demand	218 (49.2)	
**Degree**	Bachelor or diploma	386 (87.1)	
	Postgraduate degree	57 (12.9)	
**Workplace**	Hospital1	174 (39.3	
	Hospital2	61 (13.8)	
	Hospital3	47 (10.6)	
	Hospital4	144 (32.5)	
	Hospital5	17 (3.8)	
**Total**	443		100

**Fig. 1:**
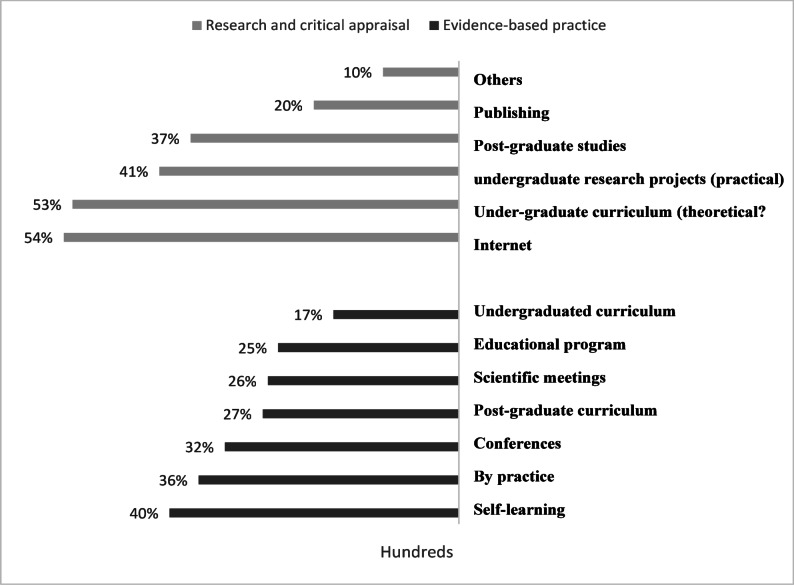
Sources of knowledge toward evidence-based practice and research and critical appraisal experience among healthcare providers in Port Said.

The most prevalent source of knowledge is self-learning (39.7%), followed by practice (35.7%), as shown in Fig. 1. Although the majority of participants had good research and critical appraisal skills (53.7% and 57.3%, respectively), 86.9% of them did not have any previous publications, as shown in Table 4.

**Table 4:** Prevalence of self-reported research and critical appraisal experiences among healthcare providers in Port said: (*N* = 443)

Variables		Frequency	Percent
**Research skills**	Good	237	53.7
	Poor	205	46.3
**Critical appraisal skills**	Good	254	57.3
	Poor	189	42.7
**Previous publishes**	Yes	58	13.1
	No	385	86.9
**Total**		443	100

Table 5 shows that the prevalence of good knowledge was 66% and that several factors are associated with knowledge, but the results of the logistic regression revealed that age (AOR = 4.231; 95% CI: 1.328–13.482; *p* = 0.015), EBP training (AOR = 0.274; 95% CI: 0.147–0.511; *p* < 0.001), profession (AOR = 0.241; 95% CI: 0.060–0.975; *p* = 0.046), and workplace (*P* = 0.002) were independently associated with good knowledge. The model correctly predicted 66.8% of the knowledge outcomes, and *P* < 0.001.

**Table 5:** Determinants of good knowledge of EBP: (*N* = 443)

Sociodemographic	Good knowledge *N* (%)	OR(CI)^#^		*P* value	AOR*	(CI 95%)	*P* value
Total	293 (66)	-		-	-	-	-
Age							
≤ 23 years	156(75.4%)	1(r)**†**		**< 0.001**	1 (r) †		**0.015**
> 23 years	134(56.8%)	2.328(1.549/3.501)			4.231	1.328–13.482	
**Sex**							
Female	183(62.9%)	1(r)		R	-		-
Male	107(70.4%)	1.403(0.920–2.139.920.139)		0.140	-	-	
**Experience Period**							
≤ 5 months	151(72.9%)	1(r)		**0.002**	1 (r)		0.122
> 5 months	138(58.7%)	1.895 (1.268/2.833)			0.470	0.181–1.224.181.224	
**EBP courses or training**							
No	166(55.9%)	1(r)		**< 0.001**	1 (r)		**< 0.001**
Yes	124(84.9%)	0.224(0.135/0.374)			0.274	0.147–0.511.147.511	
**Profession**							
dentist	2(50.0%)	1(r)		**R**	1 (r)		0.195
Physical therapist	1(100%)	2.324(0.341/15.822)		0.454	0.145	0.012–1.764.012.764	0.145
Nursing	160(59.3%)	1.013(988/1.039)		0.809	-	-	-
Pharmacist	50(84.7%)	1.147(1.012/1.3)		0.493	0.241	0.060–0.975.060.975	0.241
Doctor	77(70.6%)	0.558(0.301/1.033)		0.299	0.853	0.246–2.956.246.956	0.853
**Frequency of reading journals**							
Regular	62(66.0%)	1(r)		**R**	-		-
Occasionally	84(64.1%)	0.922 (0.529 − 1.61)		0.776	-	-	-
On demand	144(66.1%)	1(0.603 − 1.67)		0.987	-	-	-
Workplace							
Workplace (1)	112(64.4%)	1(r)		**R**	1 (r)		**0.002**
Workplace (2)	47(78.3%)	0.623(0.367/1.059)		0.232	-	-	-
Workplace (3)	19(57.6%)	1.638(0.990/2.711)		**0.021**	-	-	-
Workplace (4)	99(68.8%)	0.896(0.688/1.168)		0.445	-	-	-
Workplace (5)	9(52.9%)	1.537(0.621/3.801)		0.001	-	-	-
**Critical appraisal skills**							
Poor	253(63.4%)	1(r)		**0.003**	1 (r)		0.216
Good	37(84.1%)	1.820(1.224–2.705)			1.356	0.837–2.196.837.196	
**Previous publishes**							
No	244(63.4%)	1(r)		**0.017**	1 (r)		0.727
Yes	46(79.3%)	2.215(1.135/4.322)			1.206	0.422–3.448.422.448	
**Degree**							
Postgraduate degree	50(87.7%)		1(r)	**< 0.001**	1 (r)		0.364
Bachelor or diploma	240(62.2%)		4.345(1.19/9.839)		1.653	0.558–4.893.558.893	

*AOR Adjusted odds ratio, †r: reference, ^#^OR(CI): Odds ratio (Confidence interval)

Table 6 shows that almost all the participants had good attitudes toward EBP (96%). According to the determinants of attitude, participants with good knowledge of EBP (OR = 16.818; 95% CI: 3.813–74.173; *p* < 0.001), younger age (≤ 23 years; OR = 0.165; *p* = 0.001) and working at workplace [5] (OR = 7.541; *p* = 0.001) were significantly more likely to report a positive attitude. The results of logistic regression revealed that good knowledge was the only significant independent predictor of a positive attitude toward EBP (AOR = 26.197; 95% CI: 5.404–126.994; *p* < 0.001). The final model had excellent predictive performance, correctly classifying 95.9% of the participants and *p* < 0.001.

**Table 6:** Determinants of good attitudes toward EBP: (*N* = 443)

Sociodemographic	Attitude *N* (%)	OR(CI)^#^	*P* value	AOR*	(CI 95%)	*P* value
Total	225 (96)	-	-	-	-	-
Age						
≤ 23 years	192(92.8%)	1(r)**†**	**0.001**	1 (r) †		**0.338**
> 23 years	233(98.7%)	0.165 (0.047/0.578)		0.379	0.052–2.754	
**Sex**						
Female	279(95.9%)	1(r)	**1.000**	**-**	**-**	**-**
Male	146(96.1%)	1.047(0.385–2.846.385.846)		**-**	**-**	**-**
**Experience Period**						
≤ 5 months	191(92.3%)	1(r)	**< 0.001**	1 (r)		0.139
> 5 months	233(99.1%)	0.102(0.023/0.451)		0.204	0.025–1.672	
**Profession**						
Doctor	103(94.5%)	1(r)	**r**	**-**	**-**	**-**
dentist	4(100.0%)	1.039(1.001/1.078	0.630	**-**	**-**	**-**
Physical therapist	1(100.0%)	1.010(0.991/1.029)	0.809	**-**	**-**	**-**
Nursing	262(97.0%)	1.908(0.646/5.633)	0.235	**-**	**-**	**-**
Pharmacist	55(93.3%)	0.801(0.217/2.959)	0.937	**-**	**-**	**-**
**EBP courses or training**						
Yes	141(96.6%)	1(r)	0.633	**-**	**-**	**-**
No	284(95.6%)	1.442(0.033/3.214)		**-**	**-**	**-**
**Frequency of reading journals**						
Regular	90(95.7%)	1(r)	**r**	**-**	**-**	**-**
Occasionally	125(95.4%)	0.926(0.254/3.377)	0.907	**-**	**-**	**-**
On demand	210(96.3%)	1.167(0.343/3.973)	0.805	**-**	**-**	**-**
**Workplace**						
Workplace (1)	169(97.1%)	1(r)	**R**	1 (r)		**r**
Workplace (2)	57(95.0%)	1.468(0.584/3.689)	0.449	-	-	-
Workplace (3)	29(87.9%)	2.512(1.277/4.940)	0.023	-	-	-
Workplace (4)	144(100.0%)	0.624(0.335/1.164)	0.040	-	-	-
Workplace (5)	12(70.6%)	7.541(3.3/17.234)	0.001	-	-	-
**EBP Knowledge**						
Poor	137(89.5%)	1(r)	**< 0.001**	1 (r)		**< 0.001**
Good	288(99.3%)	16.818(3.813–74.17)		26.197	5.404–126.994.404.994	
**Research skills**						
Poor	**134(56.5%)**	**1(r)**	1.000	**-**	**-**	**-**
Good	**156(76.1%)**	**1.085(0.420/2.802)**		**-**	**-**	**-**
**Critical appraisal skills**						
Poor	381(95.5%)	1(r)	0.143	**-**	**-**	**-**
Good	44(100%)	2.181(0.829–5.735.829.735)		**-**	**-**	**-**
**Previous publishes**						
No	**370(96.1%)**	**1(r)**	**0.646**	**-**	**-**	**-**
Yes	**55(94.8%)**	**0.743(0.208/2.651)**		**-**	**-**	**-**

Table 7 shows that 66% of the participants reported good practices. Several factors were significantly associated with practice. However, positive attitudes (AOR = 15.134; 95% CI: 2.615–87.608; *p* = 0.002), good knowledge (AOR = 2.425; 95% CI: 1.457–4.038; *p* = 0.001), EBP training (AOR = 0.182; 95% CI: 0.090–0.369; *p* < 0.001), and research skills (AOR = 1.957; 95% CI: 1.129–3.391; *p* = 0.017) were strong independent predictors of EBP practice, as shown in Table 7. The model for practice correctly predicted 75.3% of the cases (*p* < 0.001).

**Table 7:** Determinants of good EBP practices: (*N* = 443)

Sociodemographic	Practice *N* (%)	OR(CI)^#^	*P* value	AOR*	(CI 95%)	*P* value
Total	293 (66)	-	-	-	-	-
Age						
≤ 23 years	147(71.0%)	1(r)†	**0.027**	1 (r) †		**412** **1.581**
> 23 years	143(60.6%)	1.593(1.070/2.372)		**1.581**	**0.530–4.722**	
**Gender**						
Female	187(64.3%)	1(r)	0.528	**-**	**-**	**-**
Male	103(67.8%)	1.169(0.771–1.772.771.772)		**-**	**-**	**-**
**Experience Period**						
≤ 5 months	147(71.0%)	1(r)	**0.021**	1 (r)		**0.570**
> 5 months	142(60.4%)	1.605(1.078/2.389)		**1.318**	**0.509–3.414**	
**Profession**						
Doctor	72(67.9%)	1(r)	R	**-**	**-**	**-**
dentist	2(50.0%)	0.473(0.064/3.498)	0.454	**-**	**-**	**-**
Physical therapist	1(100.0%)	1.014(0.987/1.041)	0.493	**-**	**-**	**-**
Nursing	168(62.2%)	0.779(0.486/1.248)	0.299	**-**	**-**	**-**
Pharmacist	45(76.3%)	1.520(0.738/3.130)	0.254	**-**	**-**	**-**
**Frequency of reading journals**						
Regular	63(67.0%)	1(r)	r	**-**	**-**	**-**
Occasionally	76(58.0%)	0.680(0.391/1.181)	0.170	**-**	**-**	**-**
On demand	151(69.3%)	1.109(0.661/1.860)	0.695	**-**	**-**	**-**
**EBP courses or training**						
No	162(54.5%)	1(r)	**< 0.001**	1 (r)		**< 0.001**
Yes	128(87.7%)	0.168(0.098/0.291)		0.182	0.090–0.369	
**Workplace**						
Workplace (1)	95(54.6%)	1(r)	r	1 (r)		**< 0.001**
Workplace (2)	42(70%)	1.006(0.877/1.155	**0.030**	-	-	-
Workplace (3)	22(66.7%)	0.977(0.587/1.628)	0.930	-	-	-
Workplace (4)	120(83.3%)	0.417(0.288/0.604)	**< 0.001**	-	-	-
Workplace (5)	6(35.3%)	2.057(0.793/5.337)	0.128	-	-	-
**Research skills**						
Poor	134(56.5%)	1(r)	**< 0.001**	1 (r)		0.017
Good	156(76.1%)	2.447(1.622–3.931)		1.957	1.129–3.391	
**Critical appraisal skills**						
Poor	257(64.4%)	1(r)	**< 0.001**	1 (r)		0.246
Good	33(75.0%)	2.642(1.767–3.951)		1.36	0.81–2.29.81.29	
**Previous publishes**						
No	242(62.9%)	1(r)	**0.003**	1 (r)		0.465
Yes	48(82.8%)	2.836(1.392/5.781)		1.507	0.502–4.523	
**Attitude**						
Negative	2(0.7%)	1(r)	**< 0.001**	1 (r)		**0.002**
Positive	288(99.3%)	16.818(3.813–74.17)		15.134	2.615–87.608	
**Degree**						
Postgraduate degree	50(87.7%)	1(r)	**< 0.001**	1 (r)		**0.525**
Bachelor or diploma	240(62.2%)	4.345(1.919/9.839)		**1.425**	**0.478–4.247**	

*AOR Adjusted odds ratio, †r: reference, ^#^OR(CI): Odds ratio (Confidence interval)

**Fig. 2:**
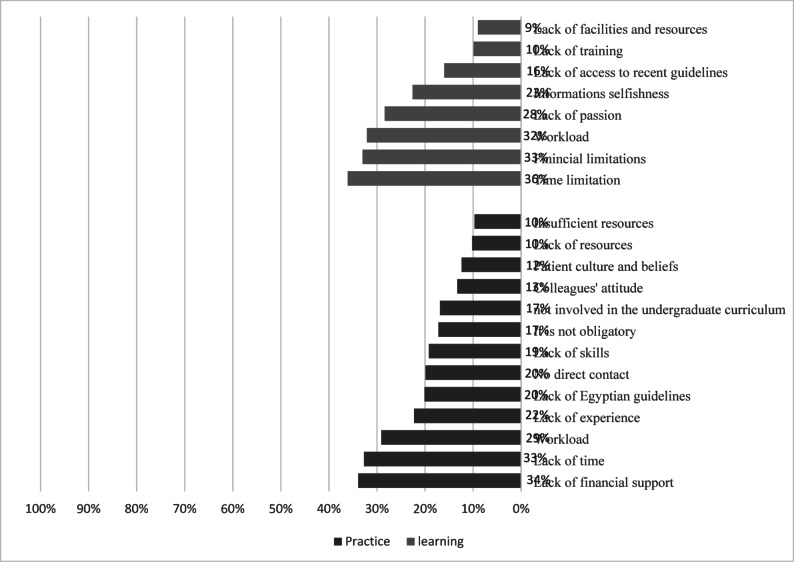
Barriers regarding the practice & learning of evidence-based practice (EBP). (*N* = 443)

Figure 2 shows the barriers to practicing and learning EBP. The most common barriers included lack of time (32.7% and 36.1%, respectively), insufficient financial support (33.9% and 33%, respectively), and workload (29.1% and 32.1%, respectively).

## Discussion

This study aimed to help improve the quality of healthcare and medical outcomes by developing and validating a questionnaire to assess the knowledge, attitudes, and practices regarding EBP among healthcare providers in Port Said city and detecting the barriers to its application after these subjects were explored. Then, they were measured quantitatively, and their associated factors were identified.

There are several validated questionnaires about EBP, but they have focused on specific populations or topics. These include the Evidence-Based Practice Questionnaire (EBPQ) for nurses; the Evidence-Based Practice Attitude Scale (EBPAS); the Fresno Test of Competence in Evidence-Based Medicine (Fresno Test), which measures knowledge and skills; the Berlin Questionnaire (EBM knowledge test); and the Knowledge, Attitude & Behavior (KAB/EBP-KABQ) questionnaires, in which the original KAB was validated for undergraduate EBP teaching [14–18]. Therefore, in the current study, a questionnaire was created to assess knowledge, attitudes, and practices regarding EBP among healthcare providers and detect barriers.

With respect to knowledge, most participants (66%) demonstrated good knowledge of EBP. This finding is relatively higher than the percentage reported in a study conducted in Tanta, Egypt, where only 10.5% of physicians had good knowledge of evidence-based medicine [12]. This attributed to the diversity of healthcare professionals included in the current study (physicians, nurses, pharmacists, etc.) as shown in a similar study in Egypt that basic EBP terms and EBP use in decision-making were mostly utilized by physicians or attributed to the increasing awareness of EBP in recent years especially in low- and middle-income countries [19, 20]. 

A similar level of knowledge was found in a Pakistani study among healthcare workers in teaching hospitals, where approximately 60% had acceptable awareness of EBP, in another study among Palestinian ICU nurses, knowledge regarding EBP was generally good, and a study in Palestine among nursing students reported that the knowledge regarding EBP was moderate (60%). In contrast, lower levels of knowledge were reported in a study from Nigeria, where only 38% of participants were familiar with EBP principles [21–24]. The differences in knowledge levels due to the variations in training opportunities, institutional support, or the availability of EBP resources in different settings. For example, the most common sources of EBP knowledge in the current study were self-learning and practice, which reflect the absence of structured formal training in most healthcare institutions. In fact, 67% of the participants in the present study reported that they had not received any formal training in EBP. These findings align with those of previous studies in Egypt and Pakistan, where the lack of training was a significant factor contributing to low EBP knowledge among healthcare providers [12, 21]. 

In the present study, several factors appeared to be associated with better knowledge and practice of EBP (P value = < 0.001). The participants who had previous training in EBP had higher knowledge scores and more positive practices, which aligns with the findings of Alqahtani et al. (2020) in Saudi Arabia on nurses, who reported that EBP-related workshops or education play a significant role in improving EBP implementation [25]. 

Additionally, an Egyptian study in Tanta on physicians revealed that job grade, specialty, and previous training concerning EBM had a statistically significant effect on knowledge scores. These findings are consistent with the current findings that age (AOR = 4.231; 95% CI: 1.328–13.482; *p* = 0.015), EBP training (AOR = 0.274; 95% CI: 0.147–0.511; *p* < 0.001), profession (AOR = 0.241; 95% CI: 0.060–0.975; *p* = 0.046), and workplace (*P* = 0.002) were independently associated with good knowledge. This indicates the need to conduct awareness campaigns, educational programs, and conferences about the importance of EBP and its application in the medical field [12]. 

Qualitative findings revealed variation in understanding: some focused on guidelines and research (“I know that it depends on the research that I learned and applying it according to its results…” R13), others integrated clinical experience (“Sometimes you are in need for balancing with a specific protocol…to save the patient’s life” R33), and a few included patient involvement (“We all assess the patient and we all share the decision with the patient. The patient also is a part of the team” R8). Participants reported learning EBP through formal education, practice, or self-directed learning, and many struggled to distinguish EBP from evidence-based medicine. These results highlight the need for standardized training and workplace support to improve both the depth and consistency of EBP knowledge.

With respect to attitudes toward EBP, almost all the participants (96%) had positive attitudes. These results are consistent with those of other studies for example, a study among Palestinian ICU nurses found generally positive attitudes toward EBP. The Palestinian study on nursing students, attitudes toward EBP were 62%, reflecting a generally positive perception of EBP and recognition of its value in clinical decision-making [22, 23]. A study conducted in Nigeria reported that 100% of primary healthcare workers had a positive attitude toward evidence-based practice, whereas a study from Syria reported that 87.4% of physicians and medical students expressed positive attitudes toward evidence-based medicine. These findings are consistent with those of a German nationwide survey, which revealed that approximately 70% of healthcare professionals had a positive attitude toward EBP [24, 26, 27]. A strong positive attitude is an indicator of the acceptance and willingness of healthcare providers for EBP application.

Additionally, healthcare providers with more years of experience tended to have slightly better attitudes and practices toward EBP, similar to the findings of Solomons and Spross (2011) [28]. However, unlike Upton et al. (2014), the present study did not find a strong association between academic qualifications and EBP knowledge, which may be due to differences in the population [29]. These variations due to differences in sample characteristics or the availability of institutional support.

The results also revealed that good knowledge was a significant predictor of a positive attitude (AOR = 26.197; *p* < 0.001). This finding agrees with the findings of a study conducted in Bangladesh, where healthcare professionals with higher knowledge levels were more likely to express positive attitudes toward evidence-based practice [30]. This confirms the interconnection between knowledge and attitudes and emphasizes the importance of enhancing knowledge to improve acceptance.

Qualitative findings explained the positive attitude. Providers noted that EBP saves time and effort: “Reducing wasted time…helping to implement the protocol and guidelines, and providing better service” (R26); enhances confidence and decision-making: “This makes the doctor confident that the decision, prescription, or medical decision will bring results to the patient” (R6); improves patient care and satisfaction: “Dealing with each patient individually…achieve a guaranteed result in the shortest time” (R26); and benefits the healthcare system: “To unify the practice and provision of healthcare services…establish patient-centered care” (R22). They also emphasized that EBP should be integrated into the medical curriculum to train competent future providers: “It will make a generation that has a better application of EBP and modern guidelines” (R10); “Because this will make the new generation more concerned about the patient’s interest and…giving him his rights” (R32).

With respect to practice, 66% of the participants demonstrated good EBP practices. This finding is consistent with a study conducted in Jordan, which reported that approximately 64% of nurses reported frequent use of EBP in clinical settings [31]. However, it is higher than the level of practice observed in a study conducted in Ethiopia, where only 35% of participants applied EBP regularly [32]. The higher practice level in the current study attributed to the presence of institutional policies in some hospitals or a higher level of awareness among younger healthcare providers who have benefited from more recent, EBP‑oriented curricula; for example, a recent study demonstrated that integrating an evidence‑based medicine curriculum into undergraduate medical education significantly enhanced students’ information literacy [33], especially since 53.3% of participants were aged 23 years or younger. The presence of supportive environments in some hospitals may have facilitated EBP implementation. On the other hand, the lack of standardized protocols in other settings may still limit full practice.

We found that positive attitudes (AOR = 15.134; 95% CI: 2.615–87.608; *p* = 0.002) and good knowledge (AOR = 2.425; 95% CI: 1.457–4.038; *p* = 0.001), EBP training (AOR = 0.182; 95% CI: 0.090–0.369; *p* < 0.001), and research skills (AOR = 1.957; 95% CI: 1.129–3.391; *p* = 0.017) were strong independent predictors of EBP practice. This finding is consistent with Egyptian studies in which knowledge and attitudes are associated with practice [12]. Additionally, Syrian and Ethiopian studies have shown that training and access to the internet and resources are associated factors [22, 32]. 

Regarding the application of EBP in practice, participants in qualitative phase reported variability in its use. Some relied on guidelines, consultant guidance, and experience: “Diagnosis and treating the cases according to acquired knowledge, guidelines, older doctors experience” (R7). Others applied EBP comprehensively, including patient involvement: “The doctor’s experience, studies, and the basics that the doctor has and the discussion with the patient, allows the patient to make the decision with you based on the Base and guideline” (R19). Most applied EBP in diagnosis and treatment: “I apply it when diagnosing and treating disease” (R31), while a minority did not routinely apply it but intended to when needed: “I don’t apply it, but there is an intention and it is possible for me to apply for it” (R29).

The most reported barriers were lack of time (32.7%, 36.1%), insufficient financial support (33.9%, 33%), and workload (29.1%, 32.1%). These findings are in line with those of multiple previous studies. A study in Kenya reported that the main barriers to EBP implementation were unreliable internet access and a heavy clinical workload [13], whereas a study in Ethiopia reported that time constraints and a lack of institutional resources were the most common challenges [32]. Similarly, in Germany, insufficient knowledge and lack of access to evidence-based resources were cited as major barriers [27].

Despite the participants’ willingness and positive attitudes, these systemic and organizational barriers still hinder the full implementation of EBP. The consistency of these barriers across different countries highlights the need for structured institutional interventions. The fact that more than 30% of the participants cited a lack of time and financial support as obstacles emphasizes the importance of managerial and governmental involvement in removing these barriers which aligns with WHO recommendations in Egypt, which emphasize fostering robust partnerships among governmental bodies, NGOs, universities, and international organizations to strengthen the health system, promote evidence-based policies, enhance healthcare workforce capacity, and improve access to services [34]. 

The qualitative findings suggest that more attention should be given to these barriers, such as establishing Egyptian guidelines for all specialties and increasing financial support for the healthcare system to improve facilities and providers, as a form of appreciation and encouragement for the application of EBP is recommended which align with the Egyptian National Health Strategy 2024–2030, which highlights strengthening institutional capacity, evidence-based protocols, and access to research resources as priorities for improving healthcare delivery by facilitating the execution of The National Strategic Plan for Health Research 2023 [35]. Additionally, hospitals must include evidence-based protocols that supervise counseling for its implementation and establish a research department that provides healthcare providers with the facilities needed to conduct research.

### Limitations

The mildly high nonresponse rate (26%) and unequal number of healthcare providers may lead to sampling and response bias, as the sample size may not be representative of all healthcare providers. This was a cross-sectional study that did not capture changes in knowledge, attitudes, practices, or barriers over time in addition to the inherent limitations of cross-sectional studies. Social desirability bias may potentially have an impact on this study since healthcare professionals may have exaggerated their EBP practices, attitudes, or knowledge in order to conform to professional standards. Even if anonymity was guaranteed, socially acceptable responses cannot be totally excluded. We recognize that the knowledge section’s Cronbach’s alpha (0.62) indicates moderate internal consistency and is below the generally recognized cutoff of 0.70. The knowledge items’ heterogeneous nature, which evaluates several subdomains rather than a single, cohesive construct, is probably the cause of this lower alpha. Despite this, expert examination verified the items’ content validity, therefore we elected to retain them to ensure comprehensive coverage of the concept. In order to improve this scale’s reliability, we also advise that it be further refined and validated in future research.

## Conclusion

Despite the barriers to learning and applying EBP, there is a strong positive attitude and reasonably good knowledge and practice. Age, profession, and EBP courses or training are important determinants of good knowledge of EBP. Good knowledge of EBP is the only determinant of a positive attitude toward EBP. EBP courses or training, research skills, good knowledge, and positive attitudes are important determinants of good EBP practices.

These results highlight the necessity of organized institutional interventions, such as focused educational initiatives, the creation of regional policies, and the setup of a conducive research environment. To improve the integration of EBP into everyday healthcare and eventually improve patient care and healthcare outcomes, it is imperative to address these organizational and structural barriers. Also, establishing EBP center that provide the training services, support research, guide the physician to apply EBP, and ensure the application of EBP will be impactful.

## Supplementary Information

Supplementary Material 1

Supplementary Material 2

## Data Availability

The datasets used and analyzed during the current study are available from the corresponding author upon reasonable request.

## Abbreviations

EBM: Evidence-based medicine
EBP: Evidence-based practice
UHI: Universal health insurance
SPSS: Statistical Package for Social Science
SD: Standard deviation
