# Supplementary material for: Assessment of knowledge, attitudes, practices, and barriers to evidence-based practice (EBP) among healthcare providers at Port Said: an exploratory sequential mixed method study
Source: BMC Med Educ. 2025 Dec 19;26:125. doi: 10.1186/s12909-025-08398-8 (PMC12825183; doi:10.1186/s12909-025-08398-8)
Supplement: Supplementary file 1 — Supplementary Material 1 [file 12909_2025_8398_MOESM1_ESM.docx]

***EBP interview guide***

**A: PRE-AMBLE (5 minutes)**

1. Self-introduction.
2. Briefings on the purpose of this discussion.
3. Collect sociodemographic data.
4. Clarify any queries.
5. Obtain written consent for the interview and audio recording.

**B: DISCUSSION PHASE (40 minutes)**

**Knowledge about EBP:**

1. What do you know about EBP?
2. Where did you learn about EBP?
3. What aspects of EBP do you find most challenging?

- Why?

1. What is the difference between EBM and EBP?

**Attitude towards EBP**

1. What is the first thing that comes to mind when you think about EBP?
2. Describe any feelings or perceptions you have about EBP in health care.
3. It is said that evidence-based practice improves patients’ care! What is your opinion?

- How?

1. Do you think EBP is important for healthcare professionals learning?

- If yes, tell me more about it.
- If no, why is that?

1. What did you find most useful about EBP learning and practice? Why? Tell me more
2. Do you think EBP should be taught in medical school?
3. If yes, why?
4. If no, why?

**Practice & Barriers of EBP**

1. Do you apply EBP in your daily health practice? If not do you intend to use it?
2. Do you find it easy to search for clinical information while on duty at the hospital?

- If yes, what are the facilitators?
- If no, what are the barriers?
  - what can be done?

1. What is your previous experience in research?
2. Describe your critical appraisal skills especially.
3. What are the benefits do you think for EBP?

- For healthcare providers.
- For patients.

1. Can you tell me about the challenges or problems that you encountered in your previous EBP learning and practice?
