# Supplementary material for: Assessment of knowledge, attitudes, practices, and barriers to evidence-based practice (EBP) among healthcare providers at Port Said: an exploratory sequential mixed method study
Source: BMC Med Educ. 2025 Dec 19;26:125. doi: 10.1186/s12909-025-08398-8 (PMC12825183; doi:10.1186/s12909-025-08398-8)
Supplement: Supplementary file 2 — Supplementary Material 2 [file 12909_2025_8398_MOESM2_ESM.docx]

**Table (1): Validated questionnaire to assess the knowledge, attitude, and practice of EBP among healthcare providers.**

| **Sociodesocio-demographic characteristics** | | | | | | |
| --- | --- | --- | --- | --- | --- | --- |
| **Age: -** | | | **Gender: -** | **○ Male** | | |
|  |  |  |  | **○ Female** | | |
| **What is the highest degree of education you have attained?** | | | | | | |
| **○Bachelor's degree** | **○ Doctoral** | | | **○ Internship** | | |
| **○ Master's degree** | **○ Fellowship** | | |  | | |
| **Years of experience as a general practitioner: -** | | **Frequency of reading journals: -** | | | | |
| **Years of experience as a specialist: -** | | **○ Regularly** | | | | |
| **Workplace: -** | | **○ Occasionally** | | | | |
| **Profession: -** | | **○ On Demand** | | | | |
| **○ Doctor** | | **Did you attend EBP courses or training?** | | | | |
| **○ Dentist** | | **○ Yes** | | | | |
| **○ Physical therapist** | | **○ No** | | | | |
| **○ Pharmacist** | |  | | | | |
| **○ Nursing** | |  | | | | |
| **Specialty: -** | |  | | | | |
| **○ Anesthesiology** | | **○ Obstetrics and Gynecology** | | | | |
| **○ Dermatology** | | **○ Ophthalmology** | | | | |
| **○ Diagnostic Radiology** | | **○ Pediatrics** | | | | |
| **○ General practitioner** | | **○ Psychiatry** | | | | |
| **○ Intern** | | **○ Surgery** | | | | |
| **○ Internal medicine** | | **○ Urology** | | | | |
| **○ Intensive care** | | **○ Other:** | | | | |
| **Knowledge about evidence-based practice** | | | | **True** | **False** | **Don’t know** |
| EBP involves the process of critical appraisal of research findings to make clinical decisions | | | | ○ | ○ | ○ |
| EBP can be gained through practice only. | | | | ○ | ○ | ○ |
| EBP is based on scientific evidence rather than the patient's state to make the clinical decision. | | | | ○ | ○ | ○ |
| EBP focuses on the best currently available research without considering clinical experience. | | | | ○ | ○ | ○ |
| EBP should integrate the recent guidelines, clinical experience, and patient involvement. | | | | ○ | ○ | ○ |
| **What is/are the sources of your knowledge about EBP?** | | | | | | |
| Undergraduate curriculum Postgraduate curriculum Self-learning  By practice Conferences Scientific meetings  Educational programs Others……. | | | | | | |

| **Research experience** | | | | |
| --- | --- | --- | --- | --- |
| **1-To what degree do you rate your research skills?** | | | | |
| **None** | **Poor** | **Moderate** | **Good** | **Expert** |
| **2-To what degree do you rate your critical appraisal skills?** | | | | |
| **None** | **Poor** | **Moderate** | **Good** | **Expert** |
| **3- How many papers have you published?** | | | |  |
| **4- What are your sources of research experience?** | | | | |
| ☐ Undergraduate Curriculum(theoretical) ☐ Undergraduate research projects (Practical)  ☐ postgraduate studies ☐ internet  ☐ Publishing papers ☐ Others | | | | |

| Attitude | Disagree | Neutral | Agree |
| --- | --- | --- | --- |
| EBP can be used in all medical specialties | ○ | ○ | ○ |
| EBP provides a better diagnosis and treatment plan | ○ | ○ | ○ |
| EBP facilitates more effective communication and strengthens the doctor‒patient relationship | ○ | ○ | ○ |
| I think traditional practice is more effective than EBP | ○ | ○ | ○ |
| EBP saves provider’s time | ○ | ○ | ○ |
| EBP reduces the provider's workload | ○ | ○ | ○ |
| EBP minimizes healthcare services costs | ○ | ○ | ○ |
| EBP has a positive impact on the provider’s reputation | ○ | ○ | ○ |
| EBP enhances communication among healthcare providers and facilitates teamwork | ○ | ○ | ○ |
| EBP increases the knowledge of providers and motivates creative thinking by expanding their minds and insight | ○ | ○ | ○ |
| EBP improves providers’ knowledge about their duties | ○ | ○ | ○ |
| EBP improves providers’ clinical practice and research skills | ○ | ○ | ○ |
| EBP improves the provider’s self-confidence | ○ | ○ | ○ |
| EBP minimizes medical errors and protects from medico-legal issues | ○ | ○ | ○ |
| EBP saves patients time and effort to obtain appropriate management. | ○ | ○ | ○ |
| EBP improves patient’s health | ○ | ○ | ○ |
| EBP improves patient’s health education | ○ | ○ | ○ |
| EBP establishes patient-centered care | ○ | ○ | ○ |
| EBP helps to provide healthcare standardization | ○ | ○ | ○ |
| The medical school systems of Egypt should include EBP in their curriculum | ○ | ○ | ○ |
| If you agree; To what extent do you agree with these? | ○ | ○ | ○ |
| The teaching of EBP will lead to better application by the new generation. | ○ | ○ | ○ |
| Providers who were early learned EBP will believe in its potential. | ○ | ○ | ○ |
| The teaching of EBP will encounter the problem of lacking Egyptian guidelines. | ○ | ○ | ○ |
| The teaching of EBP is more useful for postgraduate students. | ○ | ○ | ○ |

| **Practice** | **Yes** | **No** | **Not sure** | |  |
| --- | --- | --- | --- | --- | --- |
| I apply EBP in my clinical practice. | ○ | ○ | ○ | |  |
| I can formulate a good clinical question | ○ | ○ | ○ | |  |
| I always make the decision with the responsible team if needed. | ○ | ○ | ○ | |  |
| I always integrate the latest evidence, clinical experience, and patient's preferences | ○ | ○ | ○ | |  |
| **To what extent do you agree these barriers prevent you from practicing EBP?** | **Disagree** | **Neutral** | | **Agree** | |
| Lack of experience | ○ | ○ | | ○ | |
| Lack of time | ○ | ○ | | ○ | |
| Colleagues’ attitudes | ○ | ○ | | ○ | |
| Lack of skills | ○ | ○ | | ○ | |
| Lack of Financial support | ○ | ○ | | ○ | |
| Workload | ○ | ○ | | ○ | |
| Lack of staff awareness | ○ | ○ | | ○ | |
| Insufficient resources (Time, effort, money…. etc.) | ○ | ○ | | ○ | |
| ☐ No direct contact with the patient in some specialties like physiotherapists and pharmacists | ○ | ○ | | ○ | |
| ☐ Patient culture and beliefs | ○ | ○ | | ○ | |
| ☐ Lack of Egyptian guidelines | ○ | ○ | | ○ | |
| ☐ Lack of resources | ○ | ○ | | ○ | |
| ☐ Not involved in the undergraduate curriculum | ○ | ○ | | ○ | |
| ☐ It is not obligatory | ○ | ○ | | ○ | |
| ☐ others | ○ | ○ | | ○ | |
| **To what extent do you agree these barriers prevent you from learning EBP?** | **Disagree** | **Neutral** | **Agree** | |  |
| ☐ Time limitations | ○ | ○ | ○ | |  |
| ☐ Workload | ○ | ○ | ○ | |  |
| ☐ Lack of passion | ○ | ○ | ○ | |  |
| ☐ Selfishness of some doctors to share the information | ○ | ○ | ○ | |  |
| ☐ Lack of courses and training | ○ | ○ | ○ | |  |
| ☐ Financial limitation | ○ | ○ | ○ | |  |
| ☐ Lack of access to recent guidelines | ○ | ○ | ○ | |  |
| ☐ Lack of facilities and resources | ○ | ○ | ○ | |  |
| ☐ others | ○ | ○ | ○ | |  |
